# Supplementary material for: Darobactin B Stabilises a Lateral‐Closed Conformation of the BAM Complex in E. coli Cells
Source: Angew Chem Weinheim Bergstr Ger. 2023 May 31;135(34):e202218783. doi: 10.1002/ange.202218783 (PMC10952338; doi:10.1002/ange.202218783)
Supplement: Supplementary file 1 — Supporting Information [file ANGE-135-0-s001.pdf]

Angewandte Chemie
Eine Zeitschrift der Gesellschaft Deutscher Chemiker

Supporting Information

Darobactin B Stabilises a Lateral-Closed Conformation of the BAM Complex in *E. coli* Cells

S. F. Haysom, J. Machin, J. M. Whitehouse, J. E. Horne, K. Fenn, Y. Ma, H. El Mkami, N. Böhringer, T. F. Schäberle, N. A. Ranson, S. E. Radford, C. Pliotas**

Supplementary Information

Darobactin B Stabilises a Lateral-Closed Conformation of the BAM Complex in *E.coli* Cells

Samuel F Haysom[a], Jonathan Machin[a], James M Whitehouse[a], Jim E Horne[a], Katherine Fenn[a], Yue Ma[b,c], Hassane El Mkami[d], Nils Böhringer[e], Till F Schäberle[e,f,g], Neil A Ranson[a], Sheena E Radford*[a] and Christos Pliotas*[b,c,h]

[a] Dr. S.F. Haysom, J. Machin, Dr. J.M. Whitehouse, Dr. J.E. Horne, Dr. K. Fenn, Prof. Dr. N.A. Ranson, Prof. Dr. S.E. Radford
Astbury Centre for Structural Molecular Biology
School of Molecular and Cellular Biology
University of Leeds
LS2 9JT, Leeds, United Kingdom

[b] Dr. Y. Ma, Prof. Dr. C. Pliotas
Astbury Centre for Structural Molecular Biology
School of Biomedical Sciences
University of Leeds
LS2 9JT, Leeds, United Kingdom

[c] Dr. Y. Ma, Prof. Dr. C. Pliotas
School of Biological Sciences, Faculty of Biology, Medicine and Health, Manchester Academic and Health Science Centre
The University of Manchester
M13 9PT, Manchester, United Kingdom

[d] Dr. H. El Mkami
School of Physics and Astronomy
University of St Andrews
KY16 9SS, St Andrews, United Kingdom

[e] Dr. N. Böhringer, Prof. Dr. T.F. Schäberle
Institute for Insect Biotechnology, Natural Product Research
Justus-Liebig-University Giessen
Ohlebergsweg 12, 35392 Giessen, Germany

[f] Prof. Dr. T.F. Schäberle
Natural Product Department
Fraunhofer-Institute for Molecular Biology and Applied Ecology (IME)
Ohlebergsweg 12, 35392 Giessen, Germany

[g] Prof. Dr. T.F. Schäberle, Dr. N. Böhringer
German Center for Infection Research (DZIF), Partner Site Giessen-Marburg-Langen
Ohlebergsweg 12, 35392 Giessen, Germany

[h] Prof. Dr C. Pliotas
Manchester Institute of Biotechnology
The University of Manchester
M1 7DN, Manchester, United Kingdom

* Corresponding author(s): S.E.Radford@leeds.ac.uk or C.Piotas@leeds.ac.uk (or christos.pliotas@manchester.ac.uk)

Table of Contents

Methods *4*

Molecular biology **4**

Expression of BAM complex variants for in cell EPR **4**

Western blots of cell suspensions expressing BAM for in cell EPR **4**

Heterologous production of darobactin B **5**

In cell labelling of BAM dual-cysteine variants with MTSSL **5**

Continuous-Wave EPR (cwEPR) **6**

PELDOR distance measurements **6**

PELDOR data analysis **7**

Background correction and its impact on the modulation depth **8**

***In silico* prediction of distance distributions** **9**

Expression and purification of wild-type BAM complex for cryoEM **9**

CryoEM grid preparation **9**

CryoEM Imaging **9**

CryoEM Image Processing **10**

BAM-WT 10

BAM-WT:DAR-B 10

Model Building **10**

Supplementary Tables *11*

Supplementary Table 1 | Acquisition parameters for PELDOR experiments. **11**

Supplementary Table 2 | CryoEM data collection, refinement, validation and model building statistics. **12**

Supplementary Figures *13*

Supplementary Figure 1 | Design strategy for PELDOR spin label pairs to monitor BAM's conformation in *E. coli* cells. **13**

Supplementary Figure 2 | cwEPR results for selected spin-labelling positions on BAM. **14**

Supplementary Figure 3 | Western blots of chosen Cys-pair constructs of BAM. **15**

Supplementary Figure 4 | In cell PELDOR measurements for double-cysteine mutants of BAM. **17**

Supplementary Figure 5 | Raw PELDOR traces and distributions resulting from multiple fitting methods for L501R1-S755R1 in cells. **18**

Supplementary Figure 6 | Background corrected PELDOR data fit. **19**

Supplementary Figure 7 | PELDOR data analysis for the L501R1-S755R1 pair in the presence or absence of DAR-B. **21**

Supplementary Figure 8 | Reproducibility of in-cell EPR data. **22**

Supplementary Figure 9 | Image processing pipeline to solve the structure of BAM-WT by cryoEM...24
Supplementary Figure 10 | Image processing pipeline to solve the structure of BAM-WT:DAR-B by cryoEM……25

Supplementary Figure 11 | Comparison of apoBAM-WT and BAM-WT:DAR-B cryoEM structures determined here with existing lateral-open/closed structures of BAM. 27

Supplementary Figure 12 | Comparison of the BAM-WT:DAR-B interface with previously published structures 28

Supplementary Figure 13 | L501R1-S755R1 in cell PELDOR in presence of DAR-B is consistent with the lateral-closed state of BAM. 30

Supplementary Figure 14 | eL3 is disordered in the *apo*BAM-WT cryoEM structure solve here. 31

Supplementary Figure 15 | eL3 is poorly resolved in cryoEM structures of BAM in the lateral-open state. 32

References 33

Methods

Molecular biology

Full-length genes for BamABCDE in a pTrc99a vector, with a His_8-tag on the BamE C-terminus (pJH114)[1], was obtained from Harris Bernstein, NIH. To produce the expression plasmid used for in cell EPR work, this plasmid was modified to remove the His_8 tag from BamE, and the natural BamA cysteines were mutated to serine (BamA C690S C700S), creating Cys-free BAM. Note that BamBCDE contain only a single N-terminal Cys that is acylated and used for membrane association of these lipoproteins. Cysteines were then introduced at the desired positions in BamA. All modifications were performed using the Q5 site-directed mutagenesis kit (NEB) according to the manufacturer's instructions.

Expression of BAM complex variants for in cell EPR

All growth used carbenicillin as a selection marker. Expression plasmids for cysteine-variants of the BAM complex with the natural cysteines from BamA mutated to serine (BamA C690S C700S) were transformed into BL21(DE3) cells and plated on agar. Single colonies were then inoculated into LB and grown overnight (37°C, 200 rpm). For expression, overnight cultures were diluted 1/100 into 100 mL of 2x TY broth in baffled flasks and allowed to grow (37°C, 200 rpm) until OD_{600} reached 0.5-0.6. BAM expression was then induced with 0.4 mM IPTG and allowed to proceed for 1.5 hours, before harvesting cells by centrifugation (5,000*g*, 10 min, Beckman JA-25.50 rotor). Supernatant was discarded and cells resuspended in pre-cooled spin-labelling buffer (50 mM MOPS pH 7.5, 50 mM NaCl) supplemented with 1% (w/v) D-glucose. Resuspension volume was 3 mL buffer for every 50 mL of the original cell suspension pre-harvest. Cells were kept on ice until used for MTSSL labelling (see In cell labelling of BAM dual-cysteine variants with MTSSL).

Western blots of cell suspensions expressing BAM for in cell EPR

For western blots of whole cells, the equivalent of 1 mL of cell suspension at OD_{600} 1 was spun down (3,000*g*, 3 min) and the supernatant discarded. Cell suspensions were produced as described above, except for uninduced samples, where IPTG was not added. Pellets were either processed immediately or stored at -20 °C until needed. The pellet was resuspended in 100 µL of 1x loading dye (50 mM Tris-HCl pH 6.8, 1.5% (*w/v*) SDS, 0.001% (*w/v*) bromophenol blue, 10 % (*v/v*) glycerol) and boiled for 10 minutes (100 °C). The suspension was then spun (16,000*g*, 10 min) and 10 µL of the supernatant was loaded onto Mini-PROTEAN® TGXTM Precast Gels (Bio-Rad) and run at a constant voltage of 200 V.

Gels were transferred onto PVDF membranes using a Trans-Blot Turbo transfer system (Bio-Rad), according to the manufacturer's instructions using the 1 mini-TGX transfer protocol. Membranes were then blocked overnight in 50 mM Tris-HCl pH 7.2, 150 mM NaCl, 0.1% (*v/v*) Tween-20 (TBST), 5% (*w/v*) milk powder on a roller at 4 °C before addition of primary antibody. For detection of BamA, rabbit polyclonal antibodies raised against its C-terminal peptide (CQPFKKYDGDKAEQFQFNIGKT)[2,3] (named $\alpha BamA_{C\text{-}term}$) was used at a 1:3000x dilution in TBST, 5% *(w/v)* milk powder and allowed to

bind for 2 hours on a roller at room temperature. This polyclonal antibody was a kind gift from Harris Bernstein (NIH, USA). The membrane was then washed 3x with TBST, 5% *(w/v)* milk powder (5 min contact time per wash) before addition of anti-rabbit IgG, HRP-linked antibody (Abcam, UK) at a 1:10,000 dilution in TBST, 5% *(w/v)* milk powder. This was again allowed to bind for two hours and was then washed off with two washes of TBST and one of TBS (5 min contact time). The membrane was developed using SuperSignal^{TM} West Pico Chemiluminescent Substrate (ThermoFisher).

Heterologous production of darobactin B

For heterologous production of darobactin B, the darobactin A core peptide codons of plasmid pZW-ADC3.2[4] carrying two propeptide sequences were exchanged to WNWTKRF by PCR and subsequent recircularization in two rounds. PCR was performed using Q5 polymerase (NEB Biolabs) according to the manufacturer's instructions with the primer pair 5'-TGGAACTGGACCAAACGTTTCTAAGCATAATGCTTAAGTCGAACAG-3'/5'-GAAACGTTTGGTCCAGTTCCAAGCGGTGATTTCCGGGATTT-3'. The resulting fragment was gel purified on 1% (*w/v*) TAE agarose gels, recovered from the gel and recircularised using home-made isothermal assembly mix[5] Then, *E. coli* Top10 was transformed with the reaction using standard electroporation methodology and selected on LB supplemented with kanamycin (LB_{Kan}, final concentration 50 µg/mL). The assembled plasmid was isolated using standard miniprep methods and the process was repeated using the primer pair 5'-TGGAACTGGACGAAACGTTTCTAAAAGGAGAGCCATAAATGAATGC-3'/5'-GAAACGTTTCGTCCAGTTCCAGGCCGTGATCTCAGGGATCT-3'. Successful codon exchange was corroborated by sequencing and the final plasmid pNB-DarB3.2 was introduced to DAR resistant *E. coli* Bap1. *E. coli* Bap1 + pNB-DarB3.2 was cultivated in LB_{Kan} at 30°C and 200 rpm and darobactin B production was induced by addition of 1 mM IPTG. Darobactin B was purified from the medium according to Böhringer et al[6]. In brief, cells were separated from the medium by centrifugation after 2 days and the supernatant was extracted by addition of XAD16N resin. The resin was washed with H_2O and eluted using 50% (*v/v*) MeOH/H_2O + 0.1% (*v/v*) formic acid (FA) and the MeOH was evaporated using a rotary evaporator. The aqueous crude extract was subsequently separated using ion-exchange chromatography (SP Sepharose XL), eluting with 50 mM NH_4Ac buffer in steps with pH 5, 7, 9 and 11. Darobactin Bcontaining fraction was evaporated to dryness and final purification was performed on HPLC using a gradient of MeCN/H_2O + 0.1% (*v/v*) FA.

In cell labelling of BAM dual-cysteine variants with MTSSL

E. coli cells expressing cysteine variants of the BAM complex were prepared according to Section Expression of BAM complex variants for in cell EPR. For labelling, bacterial suspensions were diluted in Eppendorf tubes to a total volume of 1 mL and an OD_{600} reading of 10, using ice-cold spin label buffer (50 mM MOPS pH 7.5, 50 mM NaCl). In addition to the cysteine variants, each batch of samples measured included cells expressing BAM with the natural cysteines mutated out, or relevant single Cys variants, to allow measurement of background labelling. As MTSSL is rapidly reduced by the cell

suspension, with a half-life of minutes[7], timings for each labelling step were kept as similar as possible between samples to make observed signals comparable for each batch.

To each Eppendorf 1 µL of 100 mM (1-oxyl-2,2,5,5-tetramethyl-3-pyrroline-3-methyl)methanethiosulfonate (MTSSL) stock solution dissolved in DMSO was added, for a final concentration of 100 µM. Samples were placed on a thermal shaker at 25 °C and labelling allowed to proceed for 5 minutes. Cells were then pelleted (5,000*g*, 3 min, 4°C). Supernatant was discarded and the pellet resuspended in 1 mL ice-cold spin label buffer, before centrifuging again as before. For CW-EPR, the pellet was then resuspended in 20-50 µL ice-cold spin label buffer (this was kept constant for each batch of samples). A 100 µL Hamilton was then used to draw the resuspended cells into a PTFE sample loading loop (Hamilton) and load it into a 3 mm (OD) quartz tube (Wilmad® quartz (CFQ) EPR tubes, Merck) from which spectra were recorded (usually starting 17 minutes post-label).

For PELDOR, the pellet was instead resuspended in 70 µL spin label buffer to give 80 µL total volume, before mixing with 20 µL glycerol-d_8 (Sigma-Aldrich). 50 µL was then loaded into a 3 mm (OD) quartz tube (Wilmad® quartz (CFQ) EPR tubes, Merck) and flash frozen in liquid N_2.

For PELDOR measurements with and without DAR-B, pellet was resuspended in 45 µL spin label buffer. 43.9 µL of this was then mixed with 1.1 µL DAR-B ([initial] = 12 mM) and 10 µL glycerol-d_8 (Sigma-Aldrich) for a total volume of 55 µL with a final DAR-B concentration of 240 µM. 50 µL was then loaded into EPR tubes and frozen as above. For the minus DAR-B measurement, the 1.1 µL DAR-B was replaced with DMSO (solvent vehicle for the DAR-B).

Analysis of different samples with data acquired on different days shows high reproducibility of the results (once accounting for slightly different expression levels of BAM on in the different cultures (5-10 %) (Figure S8).

Continuous-Wave EPR (cwEPR)

Spectra were recorded at room temperature on a Magnettech MS-5000 (Freiburg Instruments) operating at X-band (9.2 - 9.6 GHz) using a microwave bridge power of 10 mW and 10 dB attenuation. A magnetic field sweep between 330 and 345 mT was used, with a sweep time of 60s, field modulation frequency of 100 kHz and field modulation amplitude of 0.1 mT. Spectra were averaged over 10 scans.

PELDOR distance measurements

All the pulsed and DEER/PELDOR experiments were carried out at 50K using a BRUKER ELEXSYS E580 pulsed spectrometer operating at Q-band and equipped with a Cryogen-Free Variable Temperature Cryostat (CF-VTC) from Cryogenic Ltd. The instrument is equipped with both a BRUKER 400U second microwave source unit and a SpinJet arbitrary waveform generator (AWG). The measurements were performed with a 150 W TWT Q-band amplifier and EN 5106QT-2w cylindrical resonator with typically total of 60 µL sample volume, as previously described[8,9]. Briefly, the resonator

was systematically overcoupled during all the pulsed experiments and the Q-factor was maintained approximately the same for all samples.

Echo detected field sweep (ED-FS) measurements were carried with p/2- *t* -p pulse echo detection sequence with pulse lengths set at 16 and 32 ns respectively, an inter-pulse delay of *t* = 380 ns and a shot repetition time (SRT) of 3ms.

The DEER experiments were carried out using the standard dead-time free four-pulse sequence:

p/2(*observer*)- t_1 -p(*observer*)-t--p(*pump*)- t_1+t_2-t--p(*observer*)- t_2-*echo*[10–12]

where all are rectangular pulses. The echo intensity was monitored as a function of *t*. The pump pulse was applied either using a second microwave source unit or AWG, for all experiments, at the maximum of the ED-FS spectrum whereas the observer frequency was set at 80 MHz offset from the pump frequency. The observer p/2 and p pulse lengths were set at 16 and 32 ns respectively. The p pump was set at 12 ns for maximizing the modulation depth and keeping both the observer and pump excitation bandwidths well separated. The interpulse t_1 set to 380 ns whereas t_2 was adjusted depending on the strength of the refocused echo signal and on the distances expected. The SRT was set to 3ms for all DEER experiments and the averages were typically between 6 and 46 hours until sufficient signal-to-noise was given. When using the second ELDOR source only a 2-Step phase cycling was used to remove the receiver offsets, whereas in the case of the AWG 8 step was used to remove echo crossing artifacts[13].The interpulse τ_1 was stepped eight times starting for its initial value 380 ns by 16ns and the corresponding time traces were added together to average out the deuteron modulations, which in some cases could be quite prominent in the time traces. These are typical experimental values commonly used for τ averaging to remove deuterium modulation effects during the data acquisition.

PELDOR data analysis

Distance distributions were determined from the dipolar time evolution data (experimental data) using two different MATLAB-based software programs. It was necessary to use a combination of analysis approaches given the low modulation depths, obtained in this work when using an experimental background function or a background dimensionality that deviates from the commonly used homogeneous three dimensional background (Table S1 and Fig. S6). The background correction is a major problem often met when analysing PELDOR data obtained for membrane proteins or systems which exhibit extensive aggregation phenomena due to the non-homogenous environment of the spins under study[14]. Measurements on Cys-free BAM samples had shown that the background contribution from intermolecular spins resulting from unspecific labelling of any other exposed extracellular sites of other OMPs present, fits quite well with a dimensional distribution below three (d<3). Analysis of the potential distances between BAM oligomers in the OM, such as potential BAM islands (mediated by BamB-BamB interactions[15] resulted in distances between 120 - 160 Å (with BAM in the lateral open or closed conformations), ruling out any contribution to the observed EPR data. Docking two BAM molecules together (again with BAM in lateral or closed conformations) in all possible conformations

also resulted in a low probability of complexes with distances less than 50 Å for the 501-501, 755-755 and 501-755 spin pairs (with only 0.7 % < 40 Å and 2.4 % < 50 Å). Hence non-specific oligomerisation is improbable and unlikely to affect the data analysed here.

In the present work, for the PELDOR data analysis we used two approaches, Tikhonov regularization method implemented in DeerAnalysis 2019[16] and DEERNet[17]. We used specific D values determined by fitting the background dimension when using Tikhonov regularization and the quality of the fit was assessed based on the L-curve method and the shape of the Pake pattern whereas the second method, DEERNet, doesn't require any assumption on the background dimensionality. For both methods, the distance distributions were determined with their associated uncertainties corresponding to 95% confidence intervals (2σ) obtained either by the DeerAnalysis built in validation tool or the implemented option in DEERNet that gives a measure of uncertainty of the resulting distance distributions represented by 95% confidence bounds. For the L501R1-S755R1 pair in the presence and absence of DAR-B, additionally we validate the associated results derived from these two methods by using the time trace of two control samples measured under identical conditions. These control samples used in Figure 2 for the experimentally-derived background correction were Cys-free BAM in the presence of DAR-B dissolved in DMSO (for L501R1-S755R1 in the presence of DAR-B) and Cys-free BAM in the absence of DAR-B and presence of the same final volume of DMSO used in previous condition (for L501R1-S755R1 in the absence of DAR-B). Following the experimental background model correction we performed Tikhonov regularization. Prior to the DEER data analysis, data points at the end of the time trace were cut off to remove any "2+1" end artefacts[18] appearing at the end of the PELDOR time trace.

Background correction and its impact on the modulation depth

The PELDOR data analysis for L501R1-S755R1:DAR-B and L501R1-S755R1 pairs has shown that the modulation depth is strongly dependent on the type of the background correction function (Fig. 2c,d and Figure S6). This has been previously reported[14]. Both experimental and homogeneous background correction functions (d = 2.3) resulted in similar distance distributions (Fig. S5c, S6 and S7). However, the modulation depths are quite different for both mutants, 2.8% and 1.4% for Cys-free background and 5.8% and 5.9% for homogeneous background (d = 2.3), respectively. The BAM Cys-free backgrounds yielded more reliable and well-defined Pake patterns (see the associated Pake pattern in Fig. S7), however this has impacted severely the modulation depth and resulted in a poorer signal-to-noise ratio (SNR). This confirms that the use of a Cys-free and/or single Cys mutant background correction functions are desirable especially in cases where the spin labelling efficiency is not a major issue (Figure S6). Additionally, we have tested a homogeneous three-dimensional background correction function (d=3) and the associated background corrected data are shown in Fig. S6. Although it yielded the largest modulation depth (11.6% and 7.5% for L501R1-S755R1-DAR-B and L501R1-S755R1 pairs respectively (Table S1), it resulted in multimodal distance distributions with a persistent second distance around 5nm for both mutants (Fig. S7a3 and S7b3). Their associated Pake patterns are not well defined, and their shape indicate that a part of the background is attributed to the biradical contribution[16].

In silico prediction of distance distributions

Prediction of MTSSL labelling ensembles was performed using the web server implementation of MtsslWizard[19] (MtsslSuite[20], http://www.mtsslsuite.isb.ukbonn.de). Predicted distance distributions were generated with MtsslWizard and confirmed for consistency with MMM[21].

Expression and purification of wild-type BAM complex for cryoEM

Wild type BAM (BAM-WT) was expressed and purified essentially as described previously[22]. Briefly, *E. coli* BL21(DE3) was transformed with plasmid pJH114 (expressing Bam ABCDE, with a His_8 tag on the C-terminus of BamE (BamABCDE-CT-8xHis) in a pTrc99a vector, kindly provided by Harris Bernstein[1]. Cells were grown in 2TY medium to OD_{600} = 0.5-0.6 and expression induced with 0.4mM isopropyl-β-D-thiogalactoside (IPTG) for 1.5 hours before harvesting. Total membranes were isolated and solubilised with 1 % (*w/v*) n-dodecyl-β-maltoside (DDM) using a Dounce homogenizer followed by incubation at 4 °C with rolling for 2 hours. Following ultracentrifugation of the solubilized membranes, BamABCDE-CT-8xHis was isolated from the supernatant via the His-tag on BamE using Ni-NTA. After washing, BamABCDE-CT-8xHis was eluted with 500mM imidazole, protein-containing fractions were pooled and concentrated to ~5ml, before intact BamABCDE-CT-8xHis (named hereafter BAM-WT) complexes were isolated by SEC on a HiLoad 16/600 Superdex 200 column.

CryoEM grid preparation

For the BAM-WT grids, 3 µL protein, expressed and purified as described above, at 3 mg/ml was applied to 1.2/1.3 (400 mesh) Quantifoil grids, previously glow discharged for 30 sec at 30 mA in a GloQube Plus (Electron Microscopy Sciences). For BAM-WT:DAR-B grids, BAM-WT protein was mixed with 2x molar excess of darobactin-B. 3 µL of BAM-WT:DAR-B at 3mg/ml was applied to R1.2/1.3 (300 mesh) Quantifoil grids, previously plasma cleaned for 60s using a Tergio Plasma Cleaner (PIE Scientific). Grids were blotted for 6 sec at 4 °C and >90% humidity and plunge-frozen in liquid ethane with a Vitrobot Mark IV 480 (ThermoFisher).

CryoEM Imaging

Datasets were collected on a 300 keV Titan Krios electron microscope (ThermoFisher) in the Astbury Biostructure Laboratory operated with a Falcon4 detector in counting mode. For the BAM-WT dataset, were collected using a Selectris energy filter operating with a $10e^{-}V$ slit at a nominal magnification of 165,000 yielding a pixel size of 0.74. A total of 6063 movies were collected at a nominal defocus range of -1.5 to -3.0µm and a dose rate of ~5.8 e-/pixel/s. For the BAM:DAR-B dataset (BAM-WT plus DAR-B), a total of 3732 movies were collected at a nominal magnification of 96,000x yielding a pixel size of 0.83Å with a nominal defocus range of -0.9 to -3.0µm and a dose rate of ~6.4 e-/pixel/s.

CryoEM Image Processing

BAM-WT

All processing was performed in Relion4[23], unless otherwise stated. EER-file micrographs were fractionated with 1 e-/Å^2/frame (47 frames) and motion corrected and dose-weighted. CTF parameters were estimated with CtfFind4.1[24]. 307,608 particles were initially picked with crYOLO[25] (v1.8) and 2D-classified, particles in good 2D classes (140, 519 particles) were used to train a new crYOLO model which subsequently picked 656, 080 particles. Multiple rounds of 2D classification yielded 308, 444 particles which were taken forward to 3D classification. A single high-resolution class (169, 599 particles) was refined to a 3.9 Å map. An additional low-resolution class (42 %) of the particles refined to ~15 Å with a final structure consistent with the high-resolution class. The high-resolution particles were then subjected to iterative rounds of polishing[26] in Relion and non-uniform refinement[27] in cryosparc[28] (v3.1). The final map was post-processed in Relion to 3.5 Å with a b-factor of -82 Å^2.

BAM-WT:DAR-B

All processing was performed using Relion4[23], unless otherwise stated. EER-file micrographs were fractionated with 0.85 e-/Å2/frame (41 frames) and motion corrected and dose-weighted. CTF parameters were estimated with CtfFind4.1[24]. 640, 909 particles were initially picked with crYOLO[25] (v1.8) and 2D-classified yielding 363, 304 particles which were passed to 3D classification. All high- and low-resolution classes were consistent with BAM in a closed conformation. A single high-resolution class containing 56,124 particles was refined to a 4.6 Å map. The high-resolution particles were then subjected to iterative rounds of polishing[26] in Relion and non-uniform refinement[27] in cryosparc[28] (v3.1), resulting in a final resolution of 3.3 Å. The final map was locally filtered in Relion.

Model Building

The existing, lower-resolution, BAM-WT cryo-EM structure[22] (PDB ID: 5LJO) was used as a starting point for the BAM-WT structure. Following model optimisation in ISOLDE[29] the model was passed through real-space refinement in PHENIX[30] (v1.20) with secondary structure restraints and manually refined in COOT[31,32]. Geometry was assessed using molprobity[33]. The BAM-WT:DAR-A cryo-EM structure[34] (PDB ID: 7NRI) was used as a starting point for the BAM-WT:DAR-B structure. The darobactin-A was replaced with darobactin-B from the BamA barrel in complex with darobactin-B crystal structure[6] (PDB ID: 7P1C). The model was passed through real-space refinement in PHENIX[30] (v1.20) with secondary structure restraints and manually refined in COOT[31,32]c. Geometry was assessed using molprobity[33]. For both models, model building statistics are available in Supplementary Table S2.

Supplementary Tables

Samples	Phase cycling steps	Data points	Time averaging (h)	λ*	λ_{3D}** (%)	Spin labelling efficiency*** (%)
T434R1-Q801R1	8	248	9.5	2.0	7.1	26.0+/- 2.0
E435R1-S755R1	2	265	5.5	3.0	5.1	18.5 +/- 1.5
E435R1-Q801R1	8	248	15.6	2.5	6.3	23.0 +/- 2.0
L501R1-S755R1: DAR-B	2	265	46.5	5.8	11.6	42.5 +/- 3.5
L501R1-S755R1	2	256	45.6	5.9	7.5	27.5 +/- 2.5

Supplementary Table 1 | Acquisition parameters for PELDOR experiments.

All pulse lengths were kept the same. 2-step phase cycling was used when using the second microwave source and 8-step phase cycling when using the AWG. *Modulation depth values derived from processing the primary PELDOR data with a background with dimension distribution determined by DEERnet (d = 2.3). **Modulation depth values derived from processing the primary PELDOR data with a homogeneous three-dimensional background correction. ***Spin labelling efficiency estimation and error estimation based on our experimental setup, where the pump pulse lengths are ranging between 12-16ns, the maximum modulation depths (λ_{max}) range between 25-30% for 100% spin labeling efficiency of a biradical nitroxide sample.

Supplementary Table 2 | CryoEM data collection, refinement, validation and model building statistics.

	BAM-Darobactin B (EMDB-16268) (PDB 8BVQ)	BAM-WT (EMDB-16282) (PDB 8BWC)
**Data collection and processing**		
Magnification	96k	165k
Voltage (kV)	300	300
Electron exposure (e–/Å^2)	34.7	44.8
Defocus range (μm)	-0.9 to -3.0	-1.5 to -3.0
Pixel size (Å)	0.83	0.74
Symmetry imposed	C1	C1
Initial particle images (no.)	363304	656 080
Final particle images (no.)	56124	169 599
Map resolution (Å)	3.3	3.5
0.143 FSC threshold		
Map resolution range (Å)	3.2 – 9.9	2.8-7.7
**Refinement**		
Initial model used (PDB code)	7NRI	5LJO
Model resolution (Å)	3.6	4.8
0.5 FSC threshold		0.5
Map sharpening *B* factor (Å^2)	Local sharpening	-82
Model composition		
Non-hydrogen atoms	11875	11269
Protein residues	1518	1442
Ligands	0	0
B factors (Å^2)		
Protein	197.40	-82
R.M.S. deviations		
Bond lengths (Å)	0.002	0.003
Bond angles (°)	0.573	0.695
Validation		
MolProbity score	1.69	1.42
Clashscore	7.55	7.65
Poor rotamers (%)	0.31	0.0
Ramachandran plot		
Favored (%)	95.94	98.27
Allowed (%)	4.06	1.73
Disallowed (%)	0.00	0.0

Supplementary Figures

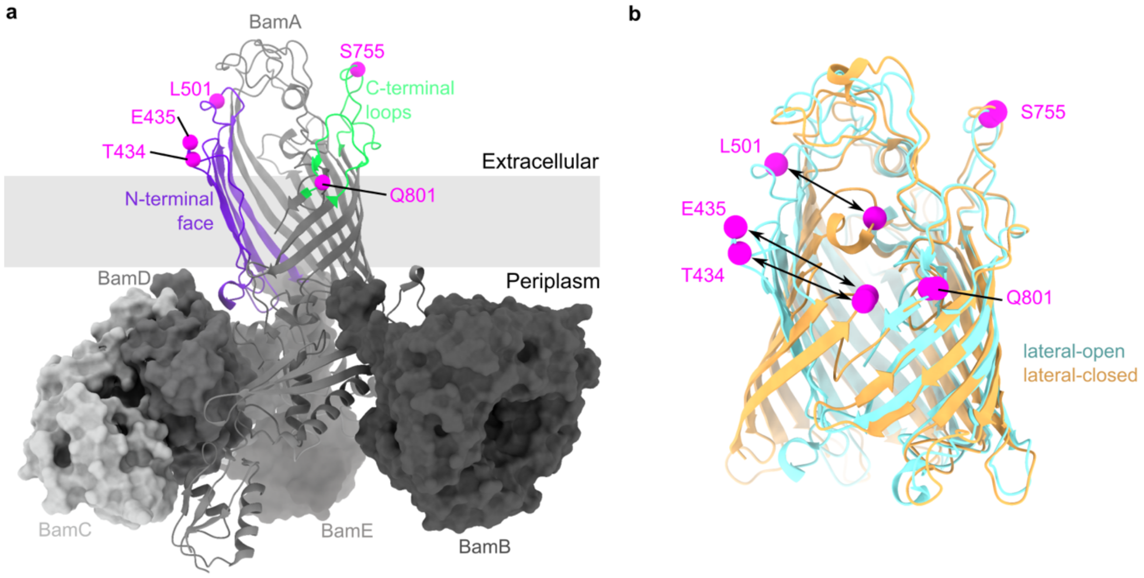

Supplementary Figure 1 | Design strategy for PELDOR spin label pairs to monitor BAM's conformation in *E. coli* cells.
a) Positions of chosen spin label positions in BamA in the intact BAM complex (PDB: 5LJO[22]). Positions for spin labelling (magenta) were chosen on the extracellular loops of BamA (which is shown in cartoon representation). Positions on the N-terminal face (purple) are mobile relative to positions on the C-terminal loops (green) between lateral-open and closed conformations of BAM, leading to distance changes. The four BAM lipoproteins, BamB-E that reside in the periplasm are shown as greyscale surfaces, and labelled. The position of the OM is shown schematically as a grey bar. **b)** Conformational change in BamA between the lateral-open (light-blue, PDB 5LJO[22]) and lateral-closed (orange, PDB 5D0O[35]) conformations of BAM. N-terminal face positions (T434, E435, L501) undergo large movements between the two conformations (arrows).

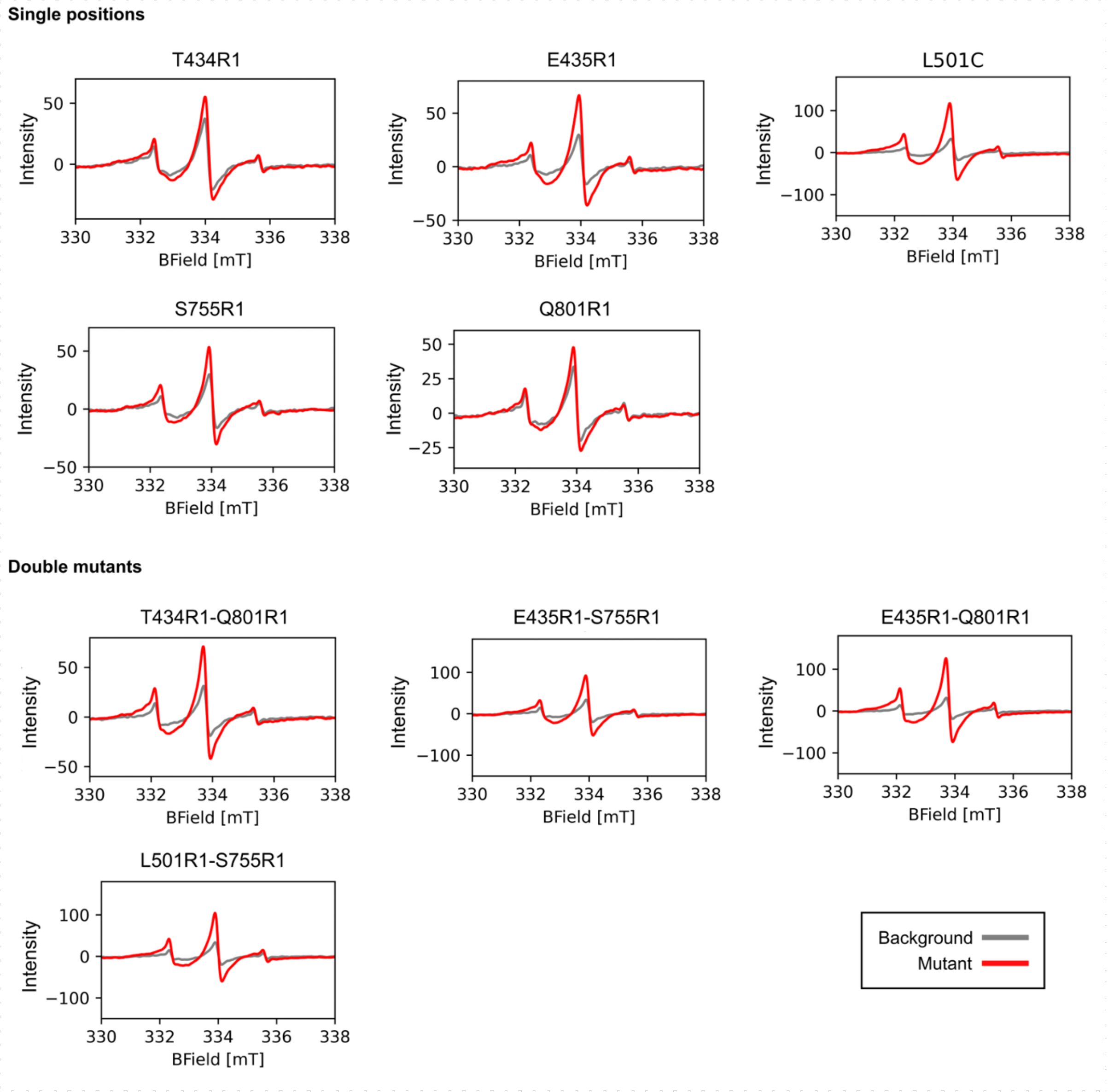

Supplementary Figure 2 | cwEPR results for selected spin-labelling positions on BAM.
The red trace indicates the cwEPR signal arising for each spin position on BAM in *E. coli* cells. The grey trace represents the signal arising from cells expressing Cys-free BAM to quantify non-specific labelling. All mutants shown presented additional spin labelling compared with background Cys-free BAM expressing cells, though to different extents, indicative of different accessibility for the different Cys introduced in BAM in the OM.

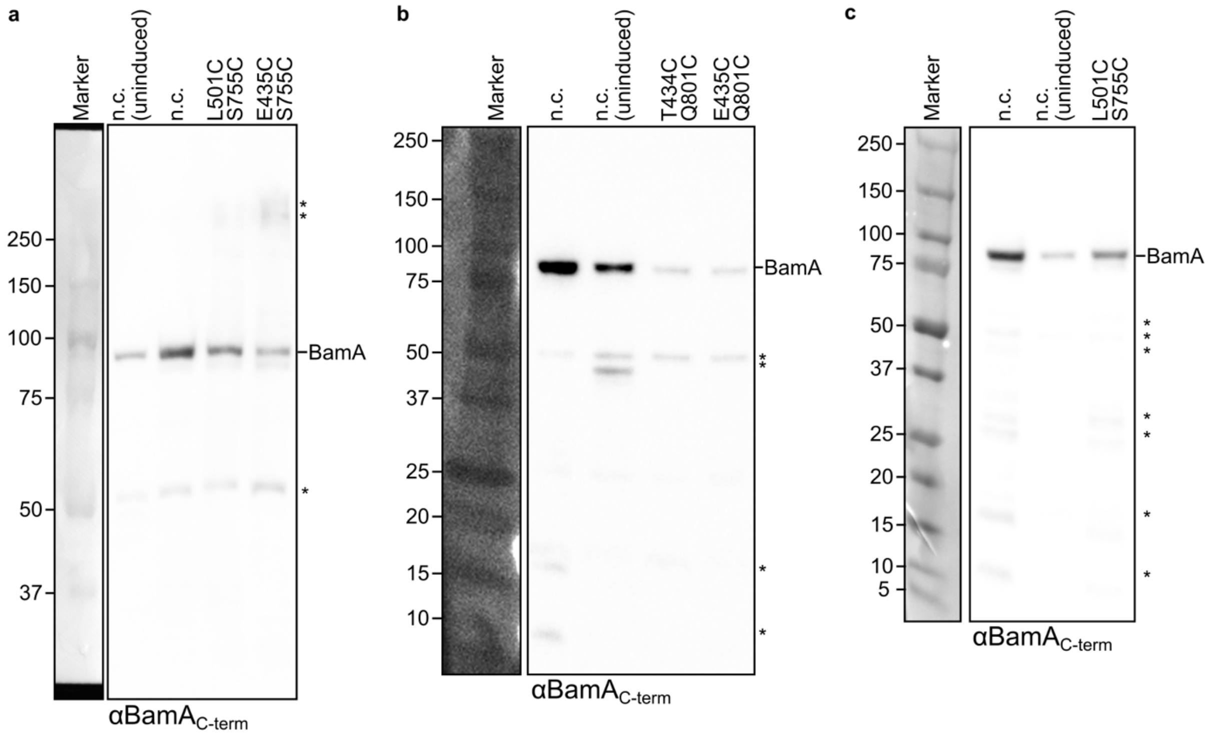

Supplementary Figure 3 | Western blots of chosen Cys-pair constructs of BAM.
Western blots of whole cells expressing selected BAM spin labelling pairs. Cell suspensions expressing each spin labelling pair were blotted against a polyclonal antibody raised against a C-terminal peptide of BamA ($\alpha BamA_{C\text{-}term}$). Shown results are for the cell suspensions used to generate the samples analysed by PELDOR of **a)** the L501C S755C and E435C S755C pairs; **b)** the T434C Q801C and E435C Q801C pairs; and **c)** the L501C S755C pair in presence/absence of DAR-B. Results for the Cys-free BAM construct used for background correction (n.c.) are also shown. Control samples where protein expression was not induced (uninduced) are indicated, showing the basal expression level from this plasmid. Possible degradation products or cross-reactive proteins are marked with an *.

a

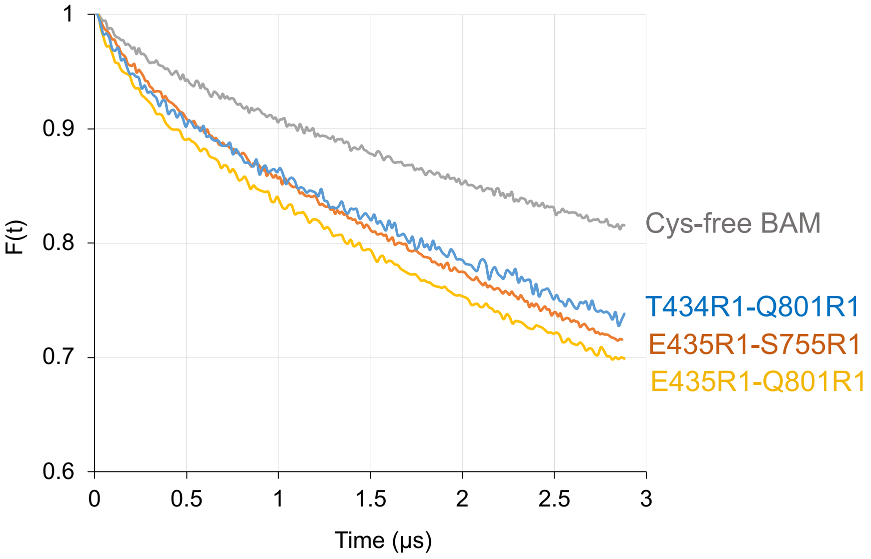

F(t)
1
0.9
0.8
0.7
0.6
0
0.5
1
1.5
2
2.5
3
Time (μs)
Cys-free BAM
T434R1-Q801R1
E435R1-S755R1
E435R1-Q801R1

b

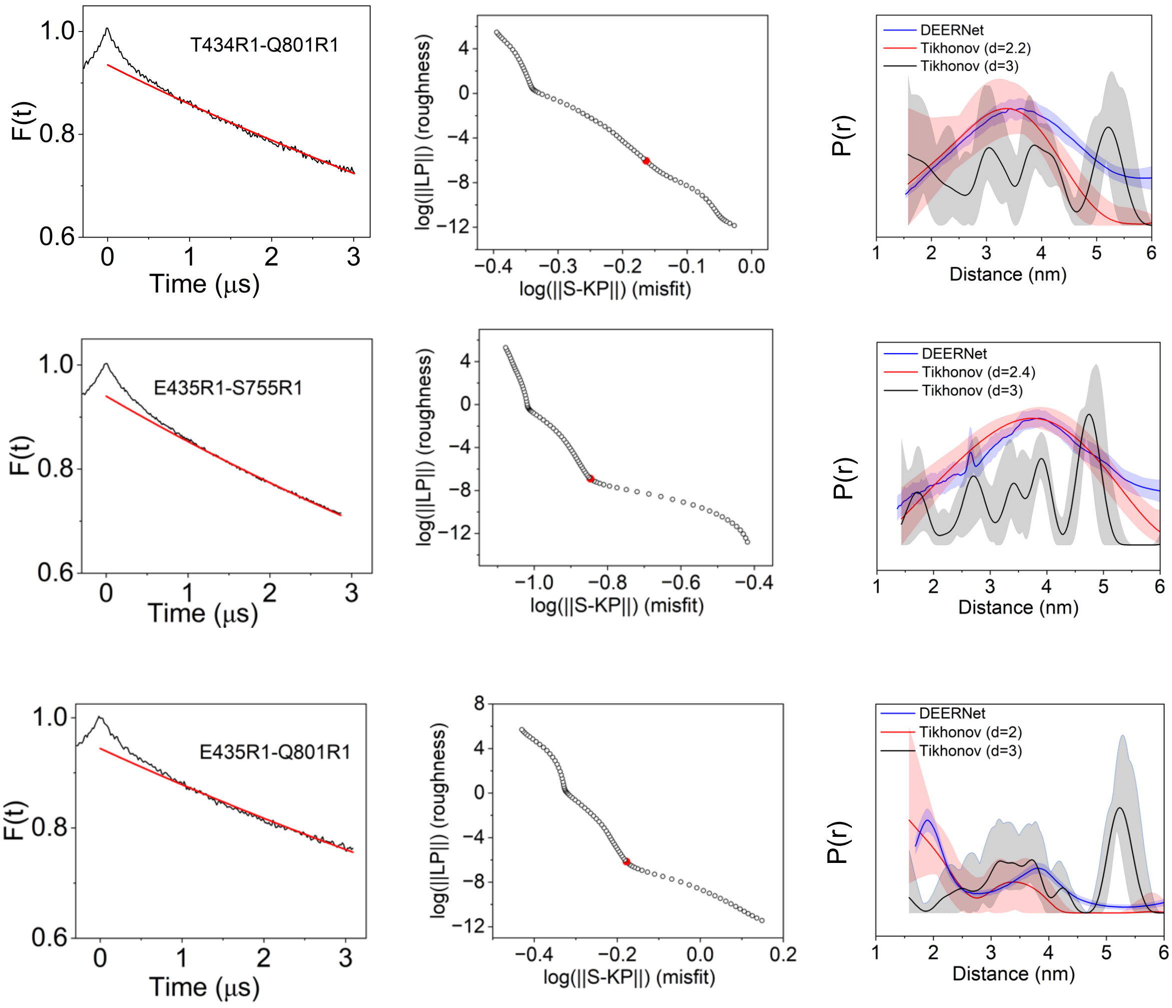

T434R1-Q801R1
E435R1-S755R1
E435R1-Q801R1
F(t)
Time (μs)
log(||LP||) (roughness)
log(||S-KP||) (misfit)
DEERNet
Tikhonov (d=2.2)
Tikhonov (d=3)
Tikhonov (d=2.4)
Tikhonov (d=2)
P(r)
Distance (nm)

Supplementary Figure 4 | In cell PELDOR measurements for double-Cys mutants of BAM.
a) Raw (uncorrected) PELDOR data traces for different BAM spin pairs and background Cys-free BAM sample. All measured BAM spin pairs deviate from the background Cys-free BAM recorded trace. Y-axis indicates PELDOR intensity. **b)** PELDOR data analysis of different double-Cys mutant pairs. Left panels, Uncorrected primary PELDOR data traces for different BAM spin pairs (black) and homogeneous three-dimensional background correction function (red). Middle panels, the associated L-curve and the corresponding regularization parameter (red dots) for the indicated spins pairs. Right panels, distance distributions obtained for the indicated pairs by using DEERNet (blue lines) and Tikhonov regularization with two different background dimensionalities (red and black lines). Y-axis for distance distributions indicates the probability density P(r). The shaded areas are error bars estimated from both methods and correspond to the variation of the probability distributions with uncertainties in the background functions. L curve is a plot of the smoothness against the means square deviation[16]. The shade areas correspond to mean ± 2σ confidence intervals of measured distributions (calculated by DeerAnalysis 2019 built-in validation tool (red and grey) or DEERNet (blue).

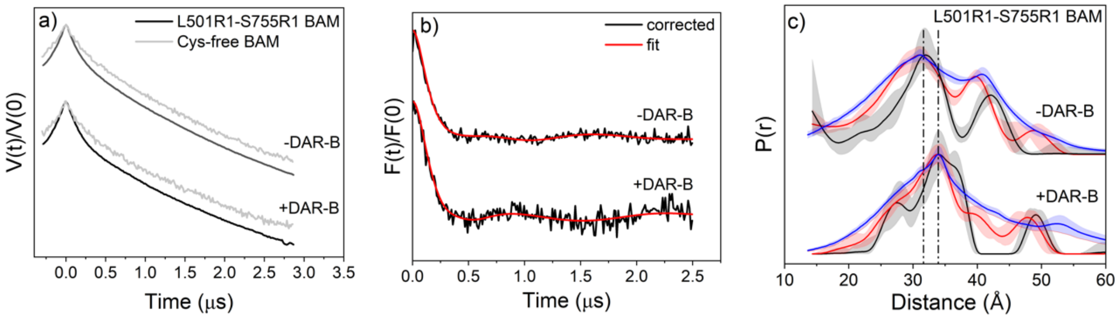

Supplementary Figure 5 | Raw PELDOR traces and distributions resulting from multiple fitting methods for L501R1-S755R1 in cells.
a) Raw (uncorrected) PELDOR traces for double mutants and control Cys-free BAM in the presence or absence of DAR-B. These respective PELDOR traces were used for experimental background-correction of the traces in **b)** Background corrected traces for 501R1-755R1 in the presence or absence of DAR-B obtained using samples expressing Cys-free BAM (Figure 2). **c)** 501R1-755R1 pair in the absence (top) or presence (bottom) of DAR-B. Distributions were obtained by performing Tikhonov regularization on time traces background corrected with the experimental control traces (grey). For further validation, we performed analysis with DEERNet (blue) and Tikhonov regularization with background dimensionality lower than 3-D (red). The shaded areas are the uncertainties estimated from each method[16,17]. Y-axis for distance distributions indicate the probability density P(r). Note, the mean values shorter than 4 nm distances remain unaffected by the different fitting background models used. Distances longer than 4 nm change dependent on the background model and fitting methods used. Thus, although some longer distances which are consistent with a BAM lateral open (eL3-out) state may exist, these were judged as not reliable based on the time windows used and were not analysed further. For assessing reliability of those distances, longer time traces should be obtained to conclude the extent of presence of the BAM lateral open (eL3-out) state in cells. However, this requires longer relaxation times, which we could not access in our experimental conditions (Figure S5).

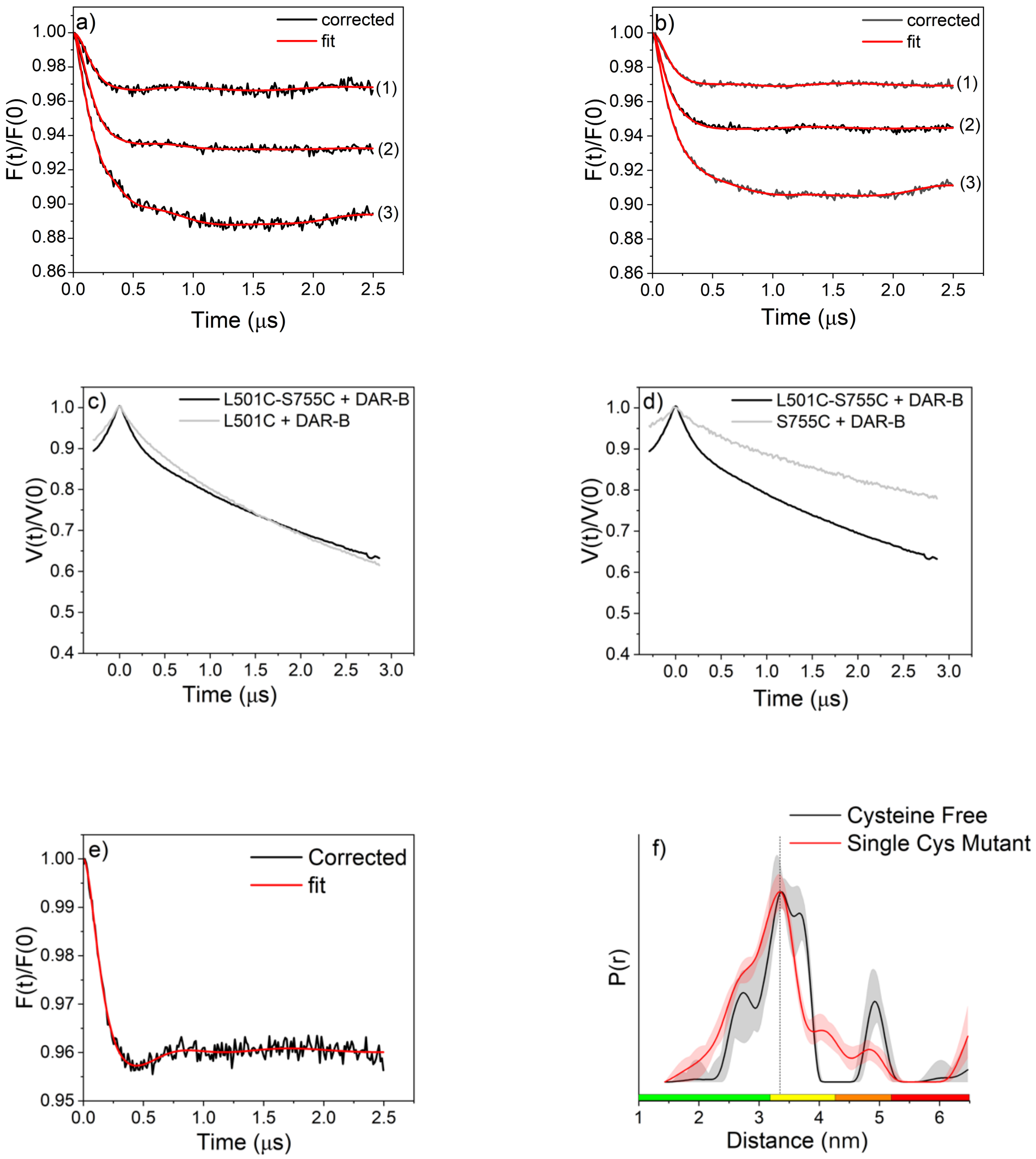

Supplementary Figure 6 | Background corrected PELDOR data fit.
a) For L501R1-S755R1 in the presence of DAR-B the PELDOR raw data were corrected with (1) experimental Cys-free BAM background function and homogeneous background function with d=2.3 (2) and d=3 (3). **b)** For L501R1-S755R1 in the absence of DAR-B the PELDOR raw data were corrected with (1) experimental Cys-free BAM background function, and homogeneous background function with d=2.3 (2) and d=3 (3). Raw data for 501R1-755R1 (black line) and **c)** single Cys 501R1(grey line) and **d)** single Cys 755R1 (grey line) both in the presence of DAR-B. **e)** Background corrected PELDOR data obtained using both single Cys (i.e. 501R1 and 755R1 weighted 1 to 1 using the DeerAnalysis tool) time traces as background. **f)** Comparison of the distance distributions obtained either by using Cys-free BAM or single Cys BAM (501R1 and 755R1) labelled mutants as experimental background.

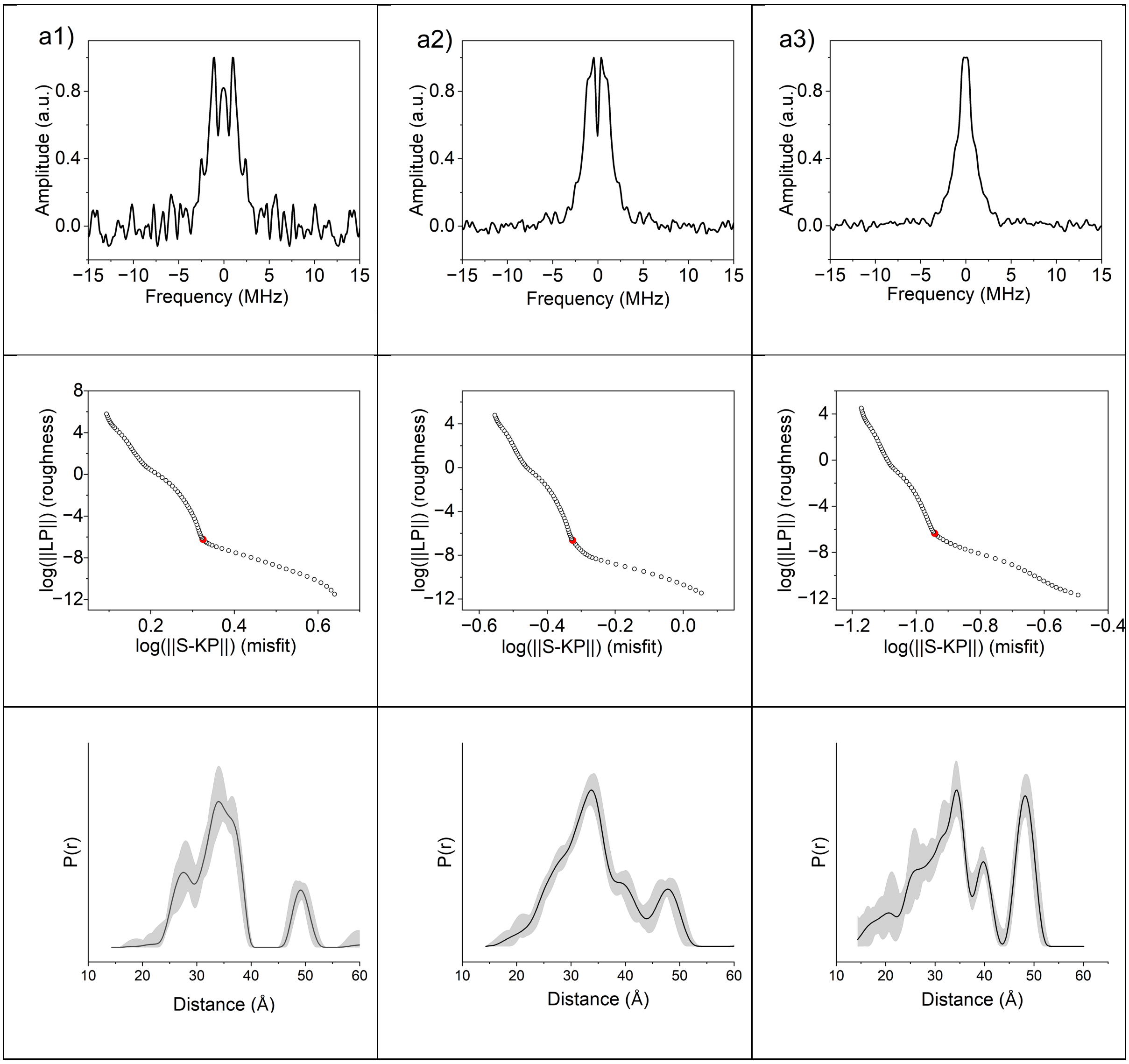
a1)
a2)
a3)
Amplitude (a.u.)
Frequency (MHz)
log(||LP||) (roughness)
log(||S-KP||) (misfit)
P(r)
Distance (Å)

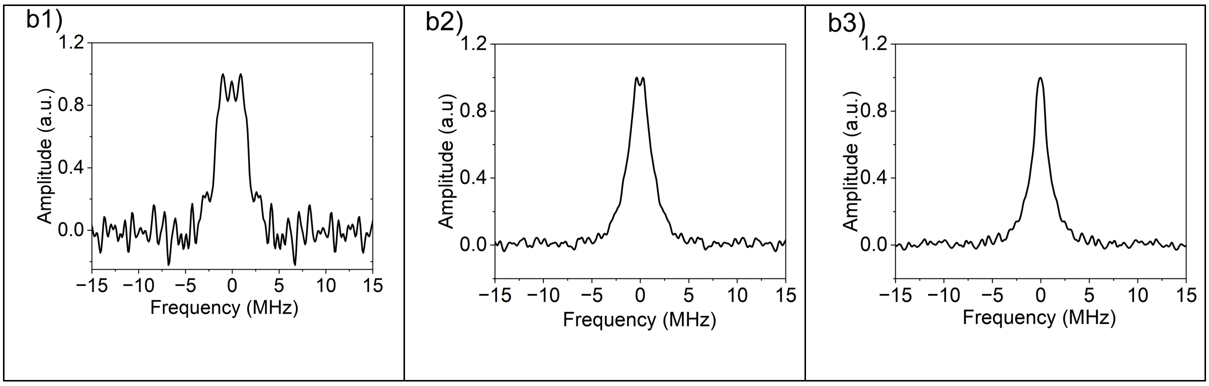
b1)
b2)
b3)
Amplitude (a.u.)
Amplitude (a.u)
Frequency (MHz)

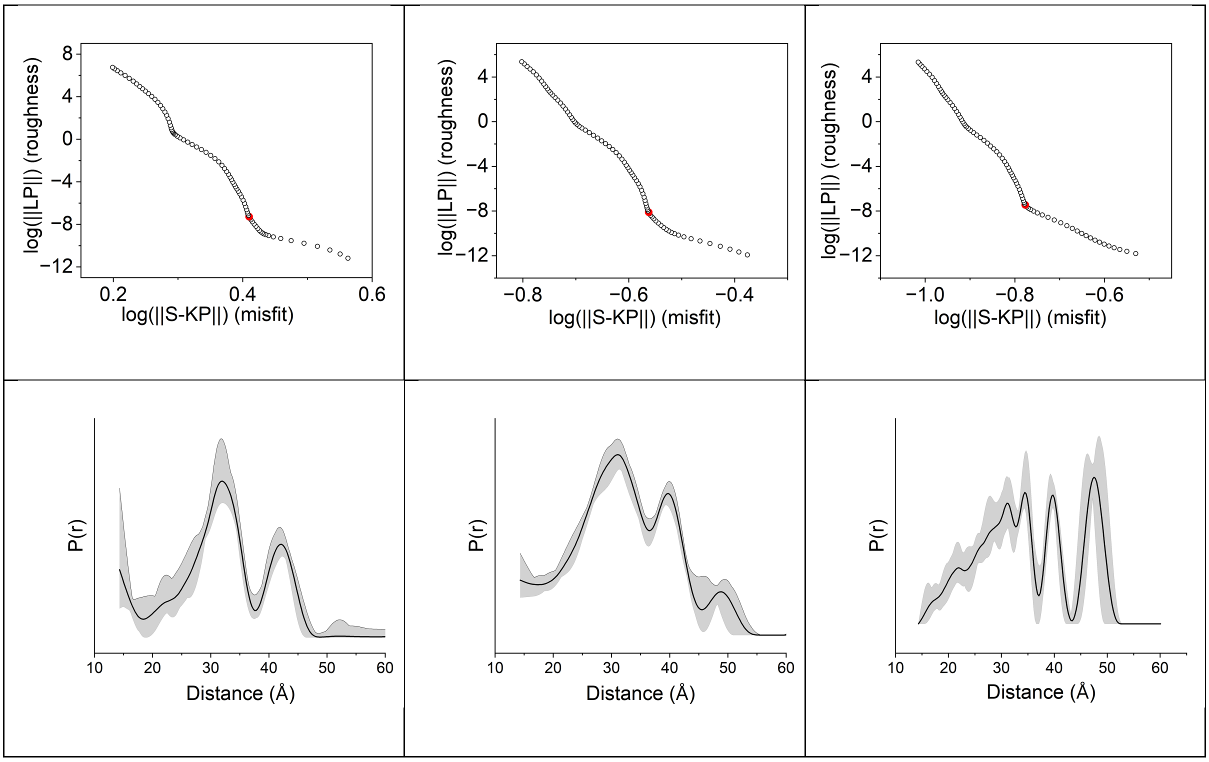

Supplementary Figure 7 | PELDOR data analysis for the L501R1-S755R1 pair in the presence or absence of DAR-B.

Data are shown in the presence **a)** or absence **b)** of DAR-B. (a1) Pake pattern, Tikhonov L-curve and distance distribution obtained by using Cys-free background function, (a2) Pake pattern, Tikhonov L-curve and distance distribution obtained by using homogeneous background function with d=2.3 and (a3) Pake pattern, Tikhonov L-curve and distance distribution obtained by using homogeneous background function with d=3. (b1) Pake pattern, Tikhonov L-curve and distance distribution obtained by using Cys-free background function, (b2) Pake pattern, Tikhonov L-curve and distance distribution obtained by using homogeneous background function with d=2.3 and (b3) Pake pattern, Tikhonov L-curve and distance distribution obtained by using homogeneous background function with d=3. The red data points in the L curve correspond to the optimum regularization parameter. L curve is a plot of the smoothness against the means square deviation [16]. Grey shade areas correspond to mean ± 2σ confidence intervals of measured distributions (calculated by DeerAnalysis 2019 built-in validation tool).

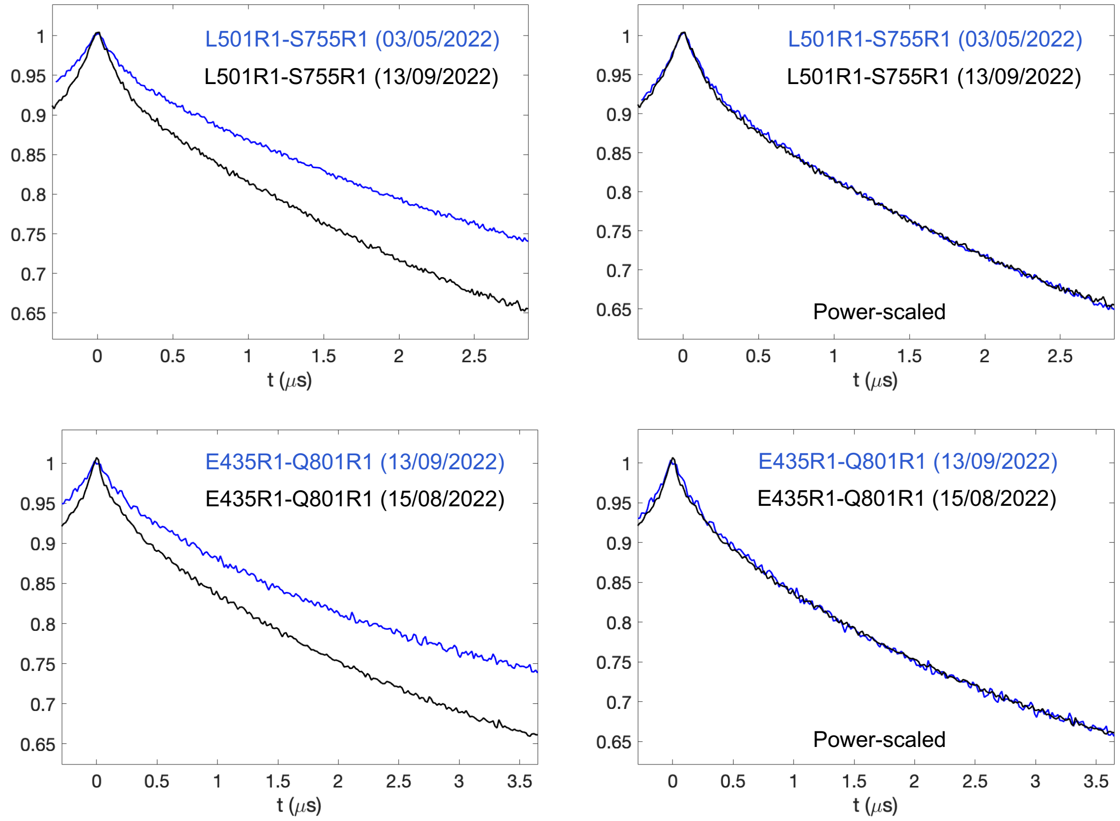

Supplementary Figure 8 | Reproducibility of in-cell EPR data.
PELDOR raw (left) and power-scaled (right) traces for the indicated spin label pairs recorded on different dates and different batches of bacterial cultures. The results portray the reproducibility of the data obtained in different biological repeats once small differences (up to ~10%) in protein expression levels in different cultures are taken into account.

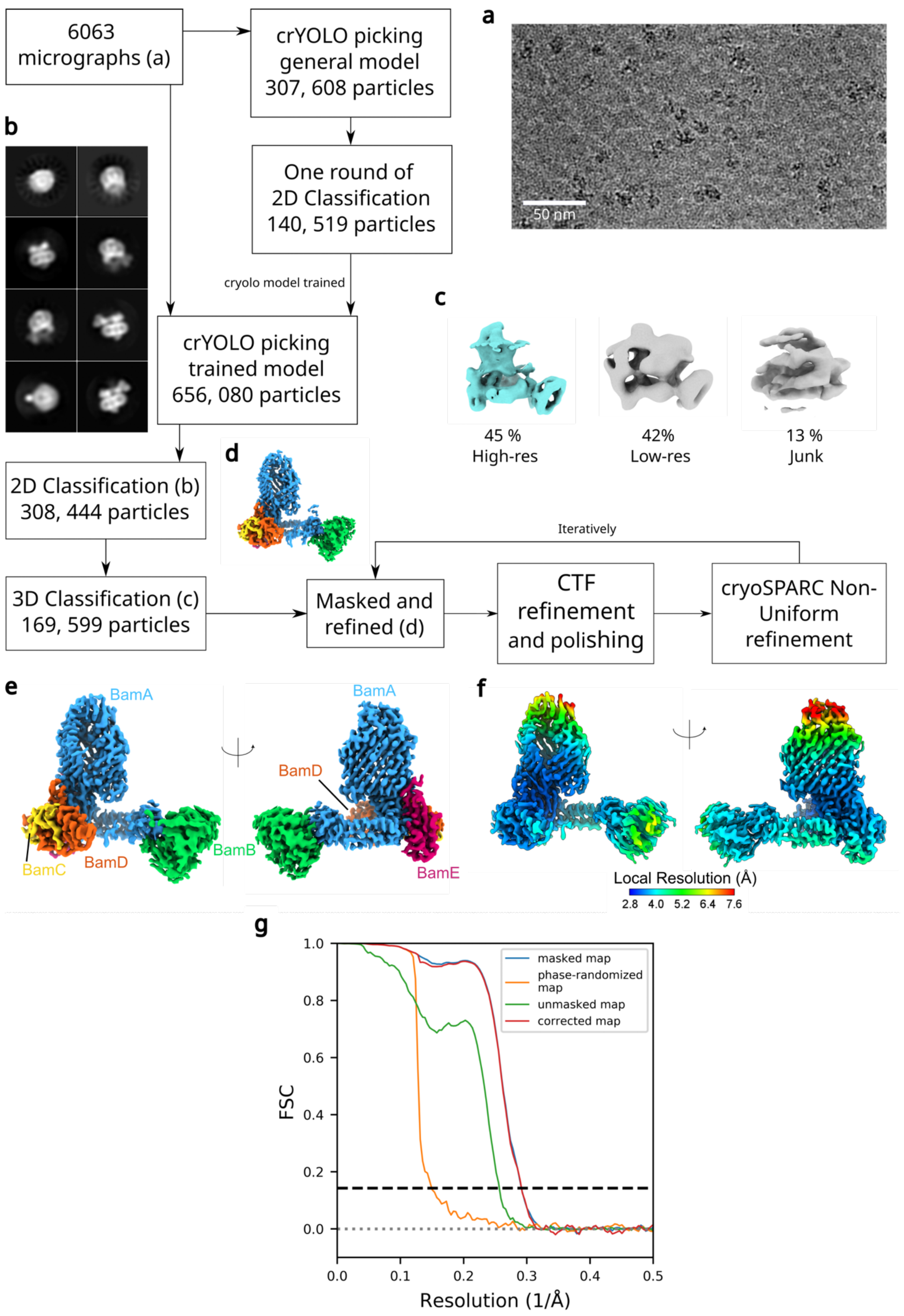
6063 micrographs (a)
crYOLO picking general model 307, 608 particles
One round of 2D Classification 140, 519 particles
cryolo model trained
crYOLO picking trained model 656, 080 particles
2D Classification (b) 308, 444 particles
3D Classification (c) 169, 599 particles
Masked and refined (d)
CTF refinement and polishing
cryoSPARC Non-Uniform refinement
Iteratively
a
50 nm
b
c
45 %
High-res
42%
Low-res
13 %
Junk
d
e
BamA
BamD
BamB
BamC
BamE
f
Local Resolution (Å)
2.8 4.0 5.2 6.4 7.6
g
FSC
Resolution (1/Å)
masked map
phase-randomized map
unmasked map
corrected map
1.0
0.8
0.6
0.4
0.2
0.0
0.0 0.1 0.2 0.3 0.4 0.5

Supplementary Figure 9 | Image processing pipeline to solve the structure of BAM-WT by cryoEM.

Image processing workflow for BAM-WT in DDM detergent. **a)** Representative raw micrograph and **b)** representative 2D classes (box size is 266.4 Å square). **c)** Particle split from 3D classification. **d)** Reconstruction after first round of refinement. **e)** Final reconstruction after iterative polishing/CTF refinement and non-uniform refinement in cryoSPARC, as in Figure S10, coloured by subunit and **f)** local resolution. **g)** FSC plot, calculated in RELION.

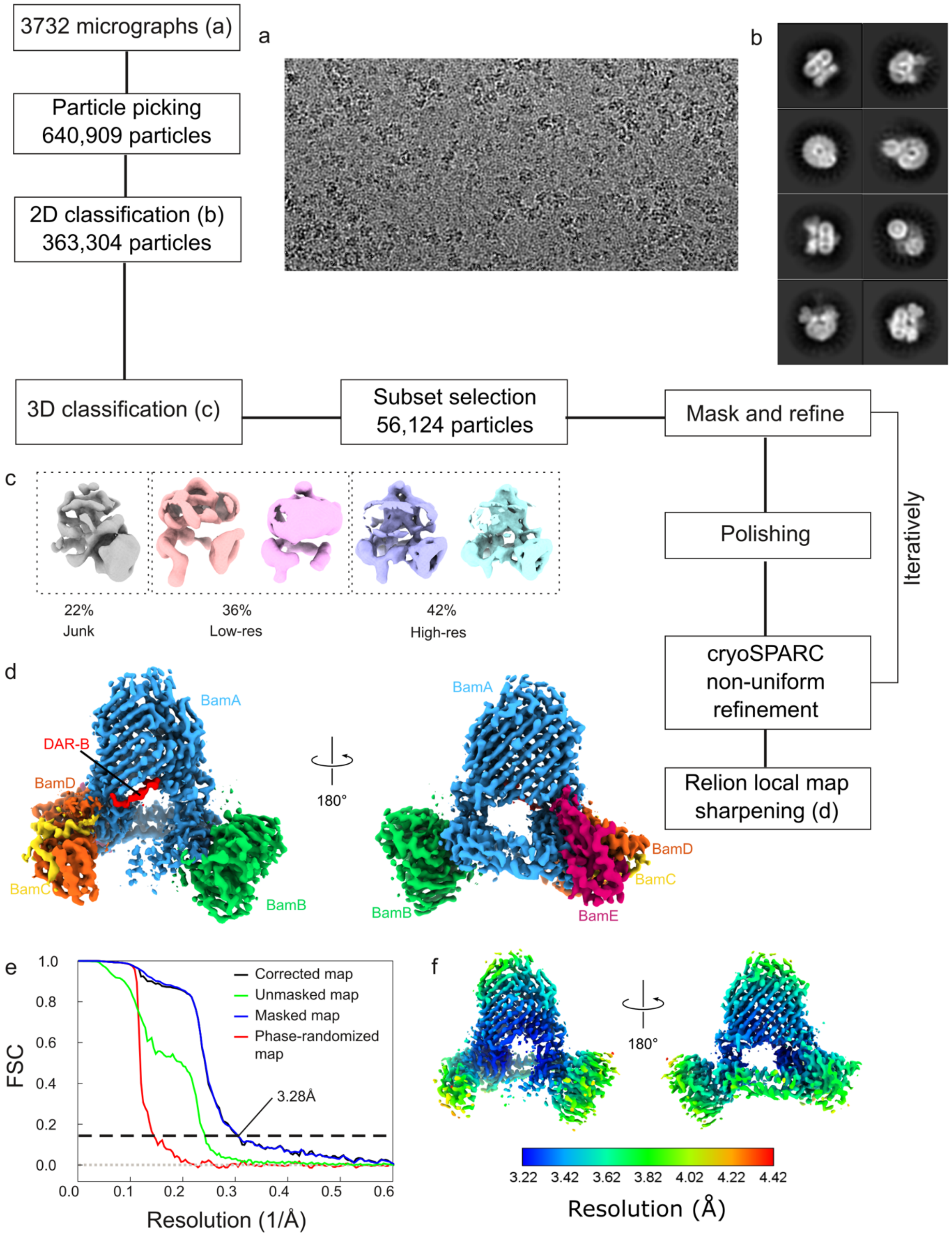

Supplementary Figure 10 | Image processing pipeline to solve the structure of BAM-WT:DAR-B by cryoEM.
Image processing workflow for BAM-WT:DAR-B in DDM detergent. Data were processed with iterative rounds of Masking, 3D refinement, and polishing in Relion imported into cryoSPARC to perform non-uniform refinement, then imported back into Relion. After the final round of refinement, a local resolution map (locally sharpened) was generated in Relion to build the model into in Phenix and Coot. **a)** Representative micrograph. **b)** Representative 2D classes (each box is 257.8 Å square). **c)** First round of 3D classification containing all particles from 2D classification. Low-res classes could not be further improved, however, they were consistent with BamABCDE and BamACDE complexes in a closed

conformation. High-res particles were taken for further downstream processing. **d)** Final map used for model building, coloured by chain. Blue = BamA, green = BamB, yellow = BamC, orange = BamD, magenta = BamE, red = darobactin B. **e)** FSC plot, calculated in Relion 4.0, used to estimate global resolution. **f)** Final reconstruction filtered and coloured by local resolution, calculated in Relion 4.0.

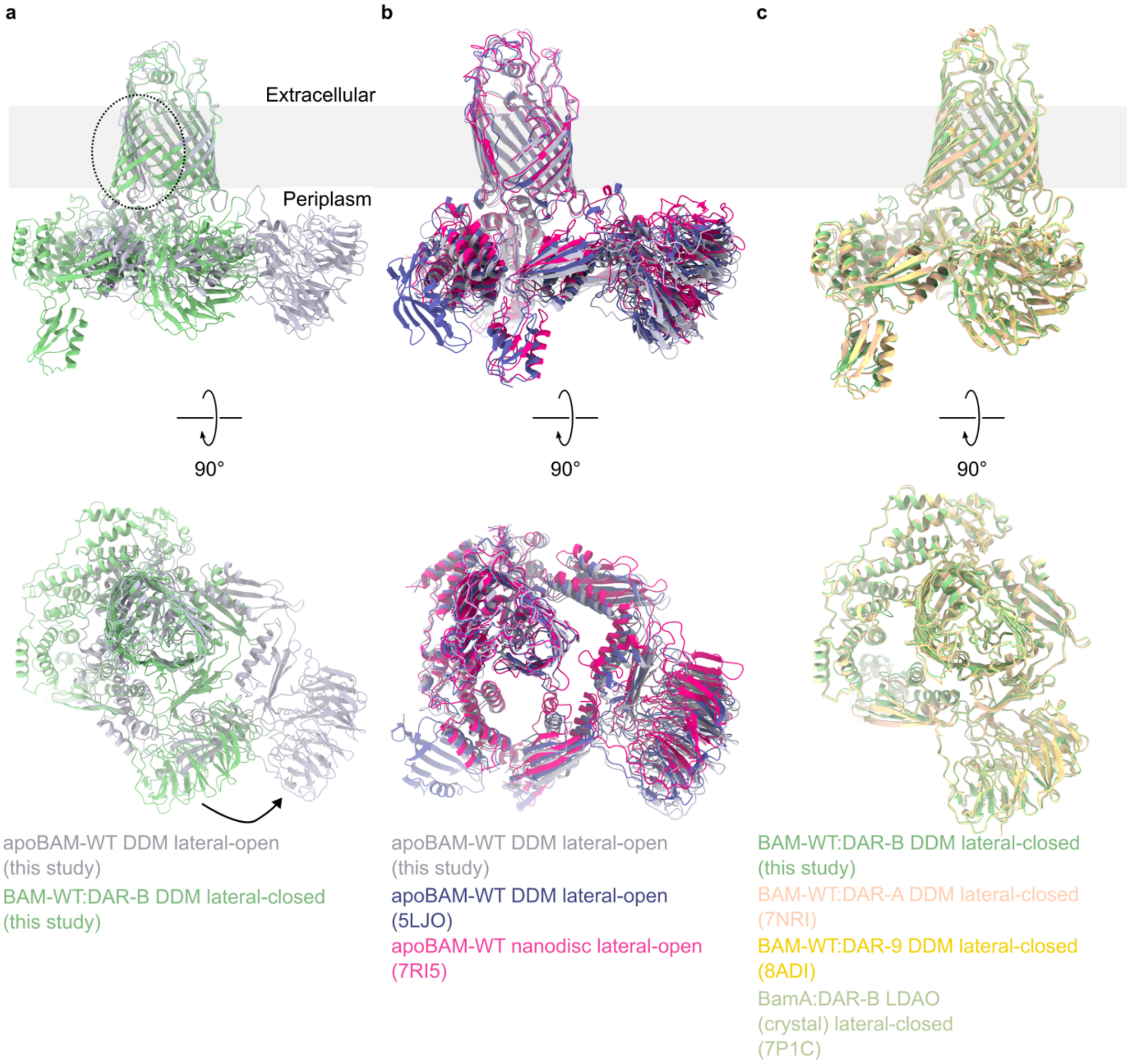

Supplementary Figure 11 | Comparison of apoBAM-WT and BAM-WT:DAR-B cryoEM structures determined here with existing lateral-open/closed structures of BAM.
In all cases, BAM structures are aligned on the back of the BamA barrel ($BamA_{600\text{-}810}$). **a)** Comparison of the lateral-open apoBAM-WT (grey) and lateral-closed BAM-WT:DAR-B (green) structures determined in this study by cryoEM. Large conformational changes are observed in the BamA barrel (circle) and in the periplasmic domains (arrow) as the lateral gate opens and closes. **b)** Comparison of the lateral-open apoBAM-WT structure determined here with previously determined cryoEM structures of BAM in a lateral-open conformation solved in DDM (PDB 5LJO[22], purple) or MSP1E3D1 nanodiscs (PDB 7RI5[36], pink), showing similarity of the structures. **c)** Comparison of the lateral-closed BAM-WT:DAR-B structure determined in this study with previously determined structure of darobactin-bound to BAM, showing the similarity of the structures. These include cryoEM structures of BAM-WT in complex with darobactin A (DAR-A) (PDB 7NRI[34], light pink) or darobactin 9 (DAR-9) (PDB 8ADI[37], yellow), both solved in DDM by cryoEM, and a crystal structure of the BamA:DAR-B complex in LDAO detergent (PDB 7P1C[6], light green). DAR-A and DAR-9 have similar binding modes to DAR-B and differ by two and three point residue changes, respectively, compared with DAR-B (DAR-B:WNWT**K**RF → DAR-B:WNW**TKRF**, DAR-A:WNW**S**K**SF**, DAR-9: WNW**S**K**SW**).

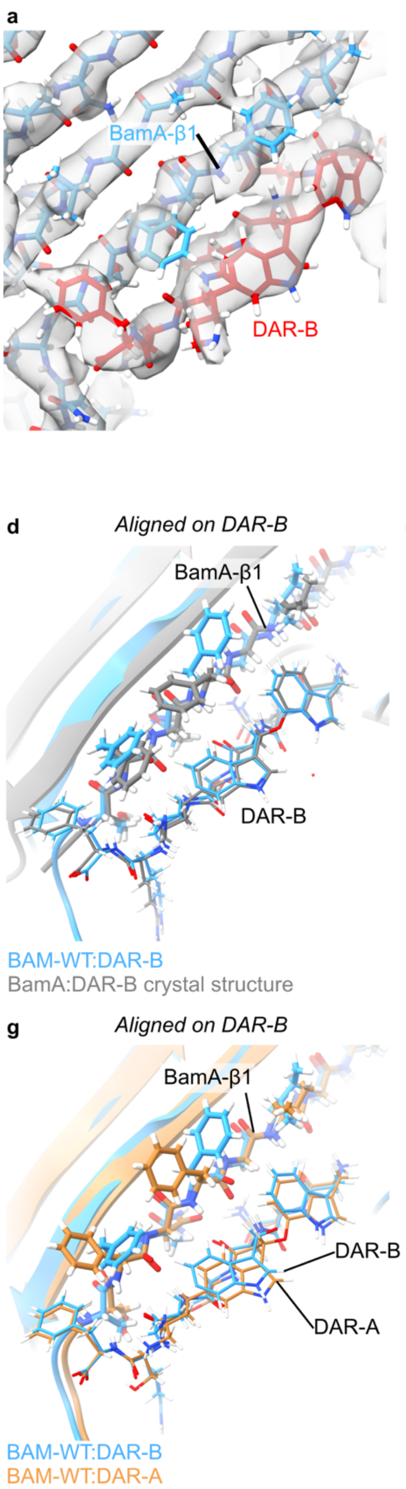

Supplementary Figure 12 | Comparison of the BAM-WT:DAR-B interface with previously published structures .

a) Map to model fit of the BAM-WT:DAR-B cryoEM structure at the BamA (blue) to DAR-B (red) interface. **b-d)** Comparison of the BAM-WT:DAR-B cryoEM structure to the previously published BamA:DAR-B crystal structure (PDB 7P1C[6]): **b)** Alignment of the structures on the BamA barrel showing the similar global fold of the barrel. **c)** Close-up of the DAR-B interface (structures again aligned on the BamA barrel). BamA-β1 and DAR-B are located slightly further towards the barrel exterior in the BAM-WT:DAR-B cryoEM structure versus the BamA:DAR-B crystal structure (arrow). **d)** Alignment of the structures on DAR-B. DAR-B's position relative to BamA-β1 is similar in both structures. **e-g)** Comparison of the BAM-WT:DAR-B cryoEM structure to a previously published structure of BAM-WT

in complex with darobactin A (DAR-A), solved by cryoEM (PDB 7NRI[34]). DAR-B and DAR-A have similar structures, differing by two amino acids (DAR-B:WNW**T**K**R**F, DAR-A:WNW**S**K**S**F). **e)** Alignment of the structures on the BamA barrel showing the similar global fold of the barrel. **f)** Close-up of the DAR-B/A interface (structures again aligned on the BamA barrel). Darobactin and BamA-β1 are in very similar positions. **g)** Alignment of the structures on DAR-B/A. Darobactin's position relative to BamA-β1 is similar in both structures.

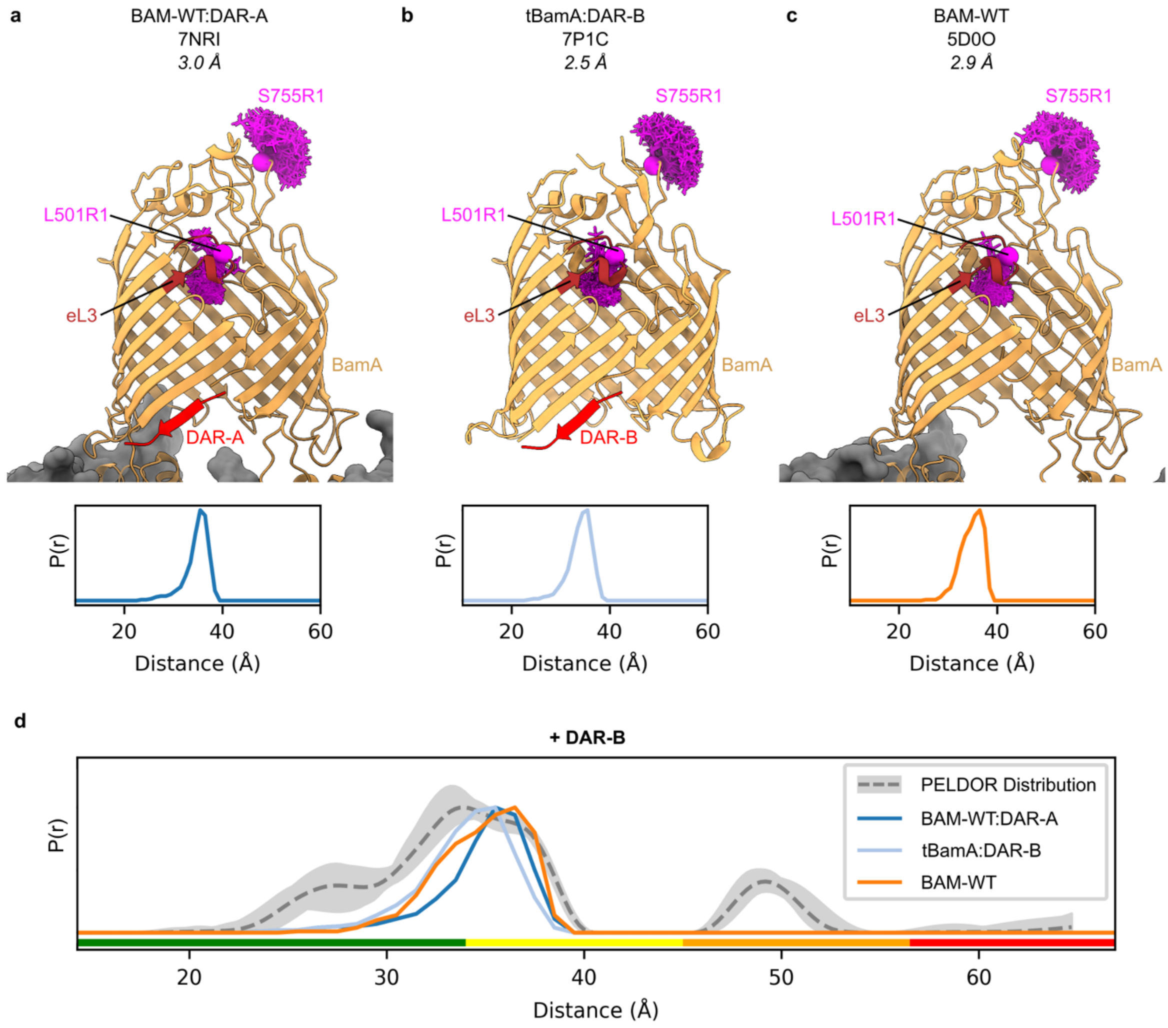

Supplementary Figure 13 | L501R1-S755R1 in cell PELDOR in presence of DAR-B is consistent with the lateral-closed state of BAM.
Lateral-closed atomic models of **a)** the BAM-WT:DAR-A complex, **b)** the BamA β-barrel (tBamA) in complex with DAR-B, and **c)** apo BAM-WT (gold) are shown, along with *In silico* generated MTSSL ensembles for L501R1-S755R1 for each structure (purple). Bottom panels for each indicate the predicted distance distribution *in silico* from MtsslWizard for each structure. **d)** Comparison of observed PELDOR distance distribution for L501R1-S755R1 +DAR-B (grey) to predicted distance distribution for the structures in **(a-c)**. For PELDOR, the mean distance is indicated by the dashed-line while shaded grey region indicates 2σ confidence intervals. Traffic light indicates reliability of PELDOR distribution: (green, shape reliable; yellow, mean and width reliable; orange, mean reliable; red, no quantification possible). Y-axis for distance distributions indicate the probability density in arbitrary units.

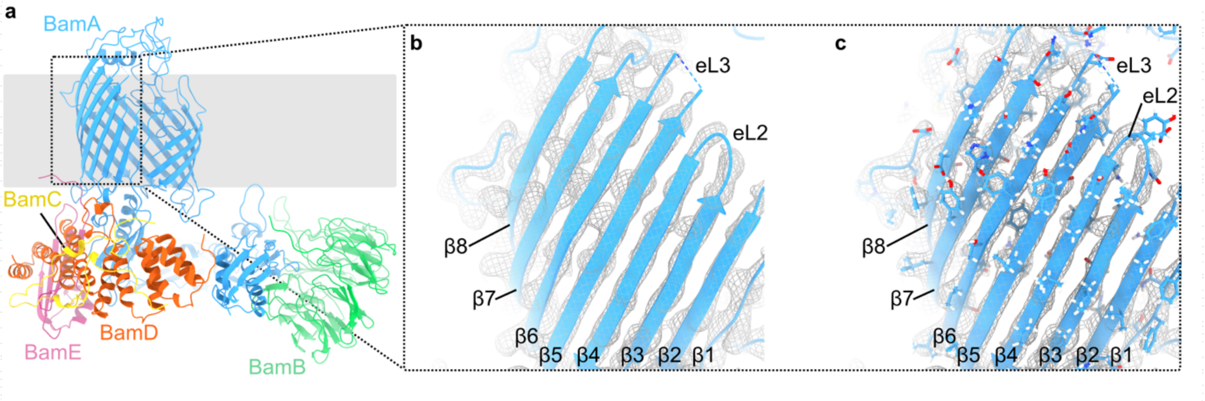

Supplementary Figure 14 | eL3 is disordered in the *apo*BAM-WT cryoEM structure solve here. **a)** Atomic model of apoBAM-WT from this study. **b)** CryoEM map and model fit for the N-terminal face of the BamA barrel expanded from the apo-BAM-WT structure. eL3 is not modelled between residues 497 and 502 due to missing density (dashed line in the structure). **c**) The same view as in **b)** but with side-chains shown to highlight model-map fit.

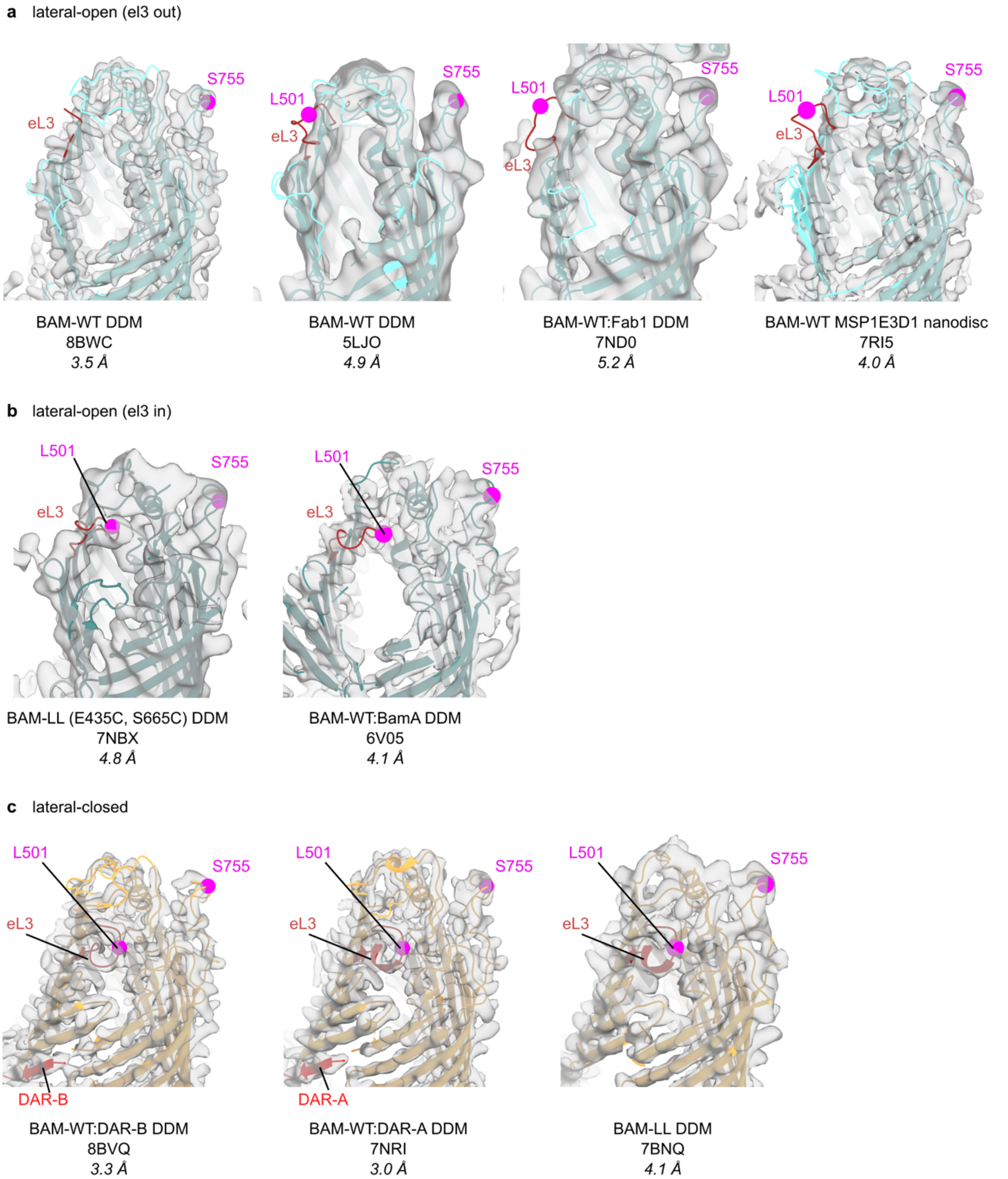

Supplementary Figure 15 | eL3 is poorly resolved in cryoEM structures of BAM in the lateral-open state.
a) CryoEM density (grey) and atomic models (light blue) for lateral-open conformations of BAM with eL3 (red) pointing out of the barrel. In all cases, density corresponding to the loop is ill-defined versus the rest of the barrel. Note that for the *apo*-BAM-WT structure determined in this paper (leftmost structure), eL3 was not modelled. **b)** CryoEM density and atomic models (dark green) for lateral-open like conformations of BAM where eL3 points into the barrel, showing the better definition of the loop in the density. **c)** CryoEM density and atomic models (gold) for lateral-closed conformations of BAM where eL3 points into the barrel. Here the loop is clearly defined in the density. DAR-A and DAR-B are shown in red where the inhibitor is present. In each figure part the position of the L501 and S755 are shown (magenta), except for the leftmost structure in **a)** where L501 is not modelled as eL3 is not resolved. PDB IDs and resolutions are indicated for each structure[6,22,34,36,38,39].

References

[1] G. Roman-Hernandez, J. H. Peterson, H. D. Bernstein, *eLife* **2014**, *3*, 04234.

[2] M. T. Doyle, H. D. Bernstein, *Nat Commun* **2019**, *10*, 1–13.

[3] O. Pavlova, J. H. Peterson, R. Ieva, H. D. Bernstein, *Proc Natl Acad of Sci of U S A* **2013**, *110*, E938–E947.

[4] Z. G. Wuisan, I. D. M. Kresna, N. Böhringer, K. Lewis, T. F. Schäberle, *Metabolic Engineering* **2021**, *66*, 123–136.

[5] D. G. Gibson, L. Young, R.-Y. Chuang, J. C. Venter, C. A. Hutchison, H. O. Smith, *Nat Methods* **2009**, *6*, 343–345.

[6] N. Böhringer, R. Green, Y. Liu, U. Mettal, M. Marner, S. M. Modaresi, R. P. Jakob, Z. G. Wuisan, T. Maier, A. Iinishi, S. Hiller, K. Lewis, T. F. Schäberle, *Microbiology Spectrum* **2021**, *9*, 1535-1521

[7] B. Joseph, E. A. Jaumann, A. Sikora, K. Barth, T. F. Prisner, D. S. Cafiso, *Nat Protocols* **2019**, *14*, 2344–2369.

[8] C. Kapsalis, B. Wang, H. El Mkami, S. J. Pitt, J. R. Schnell, T. K. Smith, J. D. Lippiat, B. E. Bode, C. Pliotas, *Nat Commun* **2019**, *10*, 4619.

[9] C. Kapsalis, Y. Ma, B. E. Bode, C. Pliotas, *Biophy. J.* **2020**, *119*, 448–459.

[10] M. Pannier, S. Veit, A. Godt, G. Jeschke, H. W. Spiess, *J. Magn. Reson.* **2000**, *142*, 331–340.

[11] R. G. Larsen, D. J. Singel, *J. Chem. Phys.* **1993**, *98*, 5134–5146.

[12] A. Milov, K. Salikhov, M. Shirov, *Fizika Tverdogo Tela* **1981**, *23*, 975–982.

[13] C. E. Tait, S. Stoll, *Phys. Chem. Chem. Phys.* **2016**, *18*, 18470–18485.

[14] S. L. Meichsner, Y. Kutin, M. Kasanmascheff, *Angew. Chem. Int. Ed.* **2021**, *60*, 19155–19161.

[15] S. D. Gunasinghe, T. Shiota, C. J. Stubenrauch, K. E. Schulze, C. T. Webb, A. J. Fulcher, R. A. Dunstan, I. D. Hay, T. Naderer, D. R. Whelan, T. D. M. Bell, K. D. Elgass, R. A. Strugnell, T. Lithgow, *Cell Rep* **2018**, *23,* 2782–2794.

[16] G. Jeschke, V. Chechik, P. Ionita, A. Godt, H. Zimmermann, J. Banham, C. R. Timmel, D. Hilger, H. Jung, *Appl. Magn. Reson.* **2006**, *30*, 473–498.

[17] S. G. Worswick, J. A. Spencer, G. Jeschke, I. Kuprov, *Sci. Adv.* **2018**, *4*, eaat5218.

[18] G. Jeschke, *Annu. Rev. Phys. Chem.* **2012**, *63*, 419–446.

[19] G. Hagelueken, R. Ward, J. H. Naismith, O. Schiemann, *Appl Magn Reson* **2012**, *42*, 377–391.

[20] G. Hagelueken, D. Abdullin, O. Schiemann, in *Methods in Enzymology* (Eds.: P.Z. Qin, K. Warncke), Academic Press, **2015**, pp. 595–622.

[21] Y. Polyhach, E. Bordignon, G. Jeschke, *Phys. Chem. Chem. Phys.* **2011**, *13*, 2356–2366.

[22] M. G. Iadanza, A. J. Higgins, B. Schiffrin, A. N. Calabrese, D. J. Brockwell, A. E. Ashcroft, S. E. Radford, N. A. Ranson, *Nat Commun* **2016**, *7*, 12865.

[23] J. Zivanov, T. Nakane, B. O. Forsberg, D. Kimanius, W. J. Hagen, E. Lindahl, S. H. Scheres, *eLife* **2018**, *7*, 42166.

[24] A. Rohou, N. Grigorieff, *J. of Struc. Bio.* **2015**, *192*, 216–221.

[25] T. Wagner, F. Merino, M. Stabrin, T. Moriya, C. Antoni, A. Apelbaum, P. Hagel, O. Sitsel, T. Raisch, D. Prumbaum, D. Quentin, D. Roderer, S. Tacke, B. Siebolds, E. Schubert, T. R. Shaikh, P. Lill, C. Gatsogiannis, S. Raunser, *Commun. Bio.* **2019**, *2*, 1–13.

[26] J. Zivanov, T. Nakane, S. H. W. Scheres, *IUCrJ* **2019**, *6*, 5–17.

[27] A. Punjani, H. Zhang, D. J. Fleet, *Nat Methods* **2020**, *17*, 1214–1221.

[28] A. Punjani, J. L. Rubinstein, D. J. Fleet, M. A. Brubaker, *Nat Methods* **2017**, *14*, 290–296.

[29] T. I. Croll, *Acta Crystallographica Section D: Structural Biology* **2018**, *74,* 519-530

[30] P. V. Afonine, B. K. Poon, R. J. Read, O. V. Sobolev, T. C. Terwilliger, A. Urzhumtsev, P. D. Adams, *Acta Crystallogr. Sect. D: Struct. Biol.* **2018**, 531-544

[31] P. Emsley, K. Cowtan, *Acta Crystallogr. Sect. D: Biol. Crystallogr.* **2004**,*74,* 2126-2132

[32] P. Emsley, B. Lohkamp, W. G. Scott, K. Cowtan, *Acta Crystallogr. Sect. D: Biol. Crystallogr.* **2010**, *66*, 486–501.

[33] V. B. Chen, W. B. Arendall, J. J. Headd, D. A. Keedy, R. M. Immormino, G. J. Kapral, L. W. Murray, J. S. Richardson, D. C. Richardson, *Acta Crystallogr. Sect. D: Biol. Crystallogr.* **2010**, 12-21

[34] H. Kaur, R. P. Jakob, J. K. Marzinek, R. Green, Y. Imai, J. R. Bolla, E. Agustoni, C. V. Robinson, P. J. Bond, K. Lewis, T. Maier, S. Hiller, *Nature* **2021**, *593*, 125–129.

[35] Y. Gu, H. Li, H. Dong, Y. Zeng, Z. Zhang, N. G. Paterson, P. J. Stansfeld, Z. Wang, Y. Zhang, W. Wang, C. Dong, *Nature* **2016**, *531*, 64–69.

[36] R. Wu, J. W. Bakelar, K. Lundquist, Z. Zhang, K. M. Kuo, D. Ryoo, Y. T. Pang, C. Sun, T. White, T. Klose, W. Jiang, J. C. Gumbart, N. Noinaj, *Nat Commun* **2021**, *12*, 7131.

[37] C. E. Seyfert, C. Porten, B. Yuan, S. Deckarm, F. Panter, C. D. Bader, J. Coetzee, F. Deschner, K. H. M. E. Tehrani, P. G. Higgins, H. Seifert, T. C. Marlovits, J. Herrmann, R. Müller, *Angew. Chem. Int. Ed.* **2023**, *62*, e202214094.

[38] P. White, S. F. Haysom, M. G. Iadanza, A. J. Higgins, J. M. Machin, J. M. Whitehouse, J. E. Horne, B. Schiffrin, C. Carpenter-Platt, A. N. Calabrese, K. M. Storek, S. T. Rutherford, D. J. Brockwell, N. A. Ranson, S. E. Radford, *Nat. Commun.* **2021**, *12*, 4174.

[39] D. Tomasek, S. Rawson, J. Lee, J. S. Wzorek, S. C. Harrison, Z. Li, D. Kahne, *Nature* **2020**, *583*, 473-478
